# Empathic responses to social targets: The influence of warmth and competence perceptions, situational valence, and social identification

**DOI:** 10.1371/journal.pone.0248562

**Published:** 2021-03-15

**Authors:** Tatjana Aue, Stephanie Bührer, Boris Mayer, Mihai Dricu

**Affiliations:** Institute of Psychology, University of Bern, Bern, Switzerland; University of Rome, ITALY

## Abstract

Accounts of empathy distinguish between cognitive (attribution of mental states to a social target) and emotional (sharing of emotions with a social target) empathy. To date, however, little is known about whether and how (interactions between) person perceptions, situational characteristics, and the observer-target relationship affect these constructs. The current study hence investigated (a) how the perceived warmth and competence of different social targets relate to both types of empathy, (b) whether there are differences in empathic responding to positive vs. negative scenarios, and (c) the impact of identification with the social targets. Eighty-nine participants rated cognitive and emotional empathy regarding four stereotypical target characters (student, elderly person, businessperson, alcoholic person) facing diverse positive and negative events. They also rated how warm and competent these characters appeared to them and how strongly they identified with the social targets. Results for cognitive and emotional empathy were partly overlapping, but demonstrated several significant differences, thereby demonstrating the need to investigate the two concepts separately. Notably, stereotypes of warmth predicted both cognitive and emotional empathic responses more strongly in desirable than in undesirable scenarios, which may relate to greater freedom of response to positive (rather than negative) social outcomes permitted by society. Our data show that scenario valence mattered even more for cognitive (than for emotional) empathy because it additionally moderated the effects of perceived competence and social identification. Finally, both cognitive and emotional empathy increased as a positive function of social identification, and social identification moderated effects exerted by perceived warmth and competence (yet differently for the two types of empathy investigated). Together, these findings speak to empathic responses arising from a complex interplay between perceptions (i.e., warmth and competence), scenario valence, and social identification.

## Introduction

“Empathy is about finding echoes of another person in yourself.”

Mohsin Hamid

According to recent conceptions in the literature [1,2], empathy represents the existence of an affective state in an observer that is isomorphic to the observed individual’s affective state. At the same time, the observer is aware that his/her own affective response has been caused by the monitored other’s response. Empathy is omnipresent in daily interactions and other social settings (e.g., interpersonal perceptions); correspondingly, it has been diligently investigated in recent years. While the number of studies conducted is impressive, there remain three major points to be addressed. First, individuals’ empathic responses to different social targets are clearly distinct [3–7], but it remains unclear as to how empathic responding relates to basic person characteristics that an observer attributes to the social targets (i.e., warmth/communion and competence/agency [8,9]). Second, little is known about whether differential empathic responding to different social targets depends on situational characteristics or whether it is reflected comparably across positive and negative scenarios. Third, testing is needed to determine whether increased identification with a social target results in increased empathic responding, as well as whether this holds independently of person and situational characteristics. The present work addresses these three aims.

Accounts of empathy most often distinguish between cognitive and affective components ([1,3,10–13]; for an additional motivational component, see [14]); hence we consider both types of empathic responding here. Cognitive empathy is defined as being_related to an individual’s capacity to adopt the perspective of another person and thus strongly depends on mentalizing abilities. Cognitive empathy hence ensures that people understand other people’s feeling states. The term emotional (or affective) empathy, in contrast, comprises the affective component of empathy and describes an observer feeling the way that the monitored other person must feel in a given scenario. A key element of emotional empathy, therefore, consists of the sharing of emotions, which makes it distinct from related concepts such as sympathy, empathic concern, and compassion (see [2], for details).

Support for a conceptual distinction between cognitive and emotional empathy is revealed in behavioral [15–17], neural [18–20], brain morphological [12,21], and genetic [22,23] findings. Although cognitive and emotional empathy are supposedly distinct (i.e., separate constructs), they are not conceptualized as independent. In fact, they have been suggested to be complementary facets of empathy ([14,18]; see [24] for an overview of different models of possible relations between cognitive and emotional empathy).

Previous evidence demonstrates that target characteristics influence the amount of empathy displayed by an observer. Specifically, earlier research has shown reduced (mostly emotional) empathy for out-group members compared with that for in-group members [25–31], which possibly relates to reduced moral processing mediated by the medial prefrontal cortex [32]. While suggestive, these observations call for further inspection. Essentially, one should ask whether definable perceivable target characteristics can be systematically linked with differences in empathy arising in the observer. Undeniably, as we noted earlier, social targets as members of different social groups are oftentimes perceived as being dramatically different in terms of both their interpersonal and their personal intentions and capabilities [8,9]. Existing theoretical models may assist with the identification of critical person characteristics that are ascribed to unknown individuals and that may have the potential to alter empathic responding in the observer.

The Stereotype Content Model (SCM; [9,33,34]), for instance, categorizes social groups along two orthogonal dimensions, namely, perceived warmth and competence (relating to Abele and Wojciszke’s [8] conceptions of communion and agency, respectively). Whereas the dimension of warmth can be conceived of as describing interpersonal intentions and capabilities attributed to a social target, the dimension of competence more closely relates to attributed personal intentions and capabilities. The four quadrants spanned by these two dimensions have been suggested to evoke qualitatively different affective experiences, attitudes, and behaviors in an observer [9,33,35–38].

The few existing studies on the SCM that touch upon empathic responding do not explicitly distinguish between cognitive and emotional empathy and include very low numbers of participants. From their task instructions, we conclude that two studies have investigated emotional empathy in response to the four quadrants of the SCM [39,40]. For undesirable events, respondents reported feeling worst toward warm but incompetent targets (eliciting pity), followed by warm and competent (pride) and cold and incompetent targets (disgust). The least emotional empathy was shown for cold but competent characters that instilled envy [39,40]. Furthermore, for desirable events, respondents reported feeling significantly less good toward cold and competent social targets (envy) than toward all other social targets, with no difference between the latter [39,40]. Together, these observations converge with other behavioral findings that suggest that seeing envied people in bad situations evokes Schadenfreude (i.e., malicious joy; [41,42]; but see [43,44] for possible moderating influences) rather than empathy. Thus, according to these studies, emotional empathy appears to be reduced for social targets perceived as high in competence but low in warmth, and increased for those targets with attributions of high warmth and low competence. By contrast, others [45] report more generally that emotional empathy in the observer correlates positively with the perceived warmth (but not competence) of a target.

Regarding cognitive empathy, there has been only preliminary functional magnetic resonance imaging and behavioral evidence [46,47], suggesting that people engage in inattention, dehumanization, and reduced mentalizing when seeing pictures of others perceived as cold and incompetent (i.e., those instilling disgust; e.g., substance abusers). We assume that such reduced social cognition may have direct consequences for an observer’s cognitive empathy.

Together, these observations suggest that the pattern of responses to the various target characters might look different for cognitive and emotional empathy. Notably, to our knowledge, no single study to date has tested both types of empathy for different combinations of perceived warmth and competence in the same study (see e.g. [14]). Therefore, remaining to be clarified is the degree to which the pattern of responses to different social targets is similar for cognitive and emotional empathy. Accordingly, in the current study, we investigated how attributions of warmth and competence relate to both cognitive and emotional empathy (**Aim 1**: **Influence of perceived warmth and competence on cognitive and emotional empathy**). Specifically, we investigated empathic responding to four stereotypical characters, each of which had been suggested to correspond to one of the four SCM quadrants spanned by the basic perceptual dimensions warmth and competence [33–38]. A student represented the warm and competent in-group of our student participants (instilling pride). The three remaining characters referred to social out-groups: an elderly person (warm, incompetent; instilling pity), a businessperson (cold, competent; instilling envy), and an alcoholic person (cold, incompetent; instilling disgust). Relying on the literature reviewed above [39–42,45–47], we expected cognitive empathy to be reduced for those perceived as low on both the warmth and the competence dimensions (here represented by the alcoholic character). Emotional empathy, in contrast, was expected to be especially reduced in targets perceived as cold but competent (here represented by the businessperson), but very high for targets perceived as warm and incompetent (here represented by the elderly person).

Moreover, we admitted the possibility of these effects being moderated by the valence of the experimental scenarios included. Research on empathy is most often based on seeing another person in an undesirable scenario (e.g., pain, setbacks, or misfortunes), which emphasizes comprehension (cognitive empathy) and sharing (emotional empathy) of negative feeling states (e.g. [48,49], but see [16,39–42,50] for an investigation of positive feeling states arising for undesirable scenarios). Less is known about (especially cognitive) empathy in desirable situations. In the present experiment, we hence assessed empathic responses in both undesirable and desirable events (**Aim 2: Comparison of empathic responses in undesirable vs. desirable situations**) in order to assess commonalities and differences in greater depth. Notably, as it has been proposed that people more commonly engage in the search for explanations regarding bad rather than good outcomes [51–53], we assumed that such differential engagement exerts differential effects on cognitive and emotional empathy in undesirable vs. desirable scenarios. Instead of being based on time-consuming, effortful searches for explanations or justifications, empathic responding to positive (rather than negative) outcomes might therefore rely more strongly on stereotypical properties of the social targets. Accordingly, we expected a stronger reliance on perceived warmth and competence when individuals rate their empathy toward the social targets for desirable compared with undesirable scenarios.

Finally, empathy is further influenced by the observer-target relationship. For instance, research has shown that empathic responses in an observer largely depend on factors such as the perceived fairness and gender of a social target [7,45,54]. Similarity and closeness to as well as familiarity with the observer are also influential [3,5,55]. Other factors determining empathic responding relate to the observer-target relationship as defined by the experimental setting (e.g., competition or cooperation, with competition leading to counter-empathic responding; [45,56]). Together, these characteristics can be assumed to determine the extent to which an observer identifies with another person. Correspondingly, we hypothesized that the level of social identification would correlate positively with both cognitive and emotional empathy (**Aim 3: Association of social identification with cognitive and emotional empathy**). We had no directed hypothesis regarding possible interactions of social identification with the two perceptual dimensions and scenario valence. The investigation of such interactions, therefore, was explorative in nature.

To address our three aims, in the current study, we examined empathic responses to four different characters (student, elderly person, businessperson, and alcoholic person) confronting 32 different desirable and undesirable scenarios. For each of these 128 experimental trials, participants reported their cognitive and their emotional empathy. The participants also rated their degree of identification (i.e., perceived similarity) with the four characters and categorized the characters according to perceived warmth and competence. From the reviewed literature, we hypothesized that (a) cognitive empathy would be reduced for targets perceived as low in both warmth and competence (i.e., interactive effects of attributed warmth and competence); (b) emotional empathy would be reduced for targets perceived as low in warmth but high in competence; and (c) emotional empathy would be increased for targets perceived as high in warmth but low in competence. Together, these latter two hypotheses are consistent with the idea that emotional empathy varies as a positive function of attributed warmth and as a negative function of attributed competence. We further expected (d) a stronger reliance on perceived warmth and competence for positive than for negative scenarios (for both cognitive and emotional empathy) and (e) increasing cognitive and emotional empathy with increasing levels of social identification.

## Materials and methods

### Participants

Eighty-nine German-speaking University of Bern undergraduate students (80 females), between 18 and 30 years of age (*M* = 22.2, *SD* = 2.36) were recruited via e-mails, flyers, and the local participant pool of the University of Bern. Participants were compensated with either course credits or monetary payment and gave informed and written consent for their participation. Experimental protocols, methods of data collection, data handling, and data analysis were approved by the local ethics committee of the University of Bern and are fully in accord with the Declaration of Helsinki [57].

### Experimental task

Participants specified their cognitive and emotional empathy for 16 desirable and 16 undesirable events (see S1 Table for a list of included events) for four different characters (student, elderly person, businessperson, alcoholic person; S1 and S2 Figs). The question for cognitive empathy asked “In your opinion, how good/bad does the depicted character feel in this specific situation?” (c.f., [58]) and therefore addressed metacognitive abilities. The question for emotional empathy, in contrast, asked “How good/bad do you feel when you see the depicted character in this specific situation?” (cf. [40]) and thus assessed affective sharing. Responses to both questions were given on continuous scales; the response format for these questions ranged from -50 (“very bad”) to 50 (“very good”). Desirable and undesirable events were matched with respect to event frequency and controllability (see S1 Table for details).

The four different characters referred to the four quadrants generated by the two dimensions, perceived warmth and perceived competence, as specified in the SCM [9,33,34]. A still animation of a student represented the in-group character for our student participant sample and was supposed to be perceived as high in both warmth and competence. The three remaining characters served as out-group characters: an elderly person (high warmth, low competence; associated with the experience of pity), a businessperson (low warmth, high competence, associated with the experience of envy), and an alcoholic person (low warmth, low competence; associated with the experience of disgust). The gender of the presented characters was matched with the gender of the participant (S1 Fig). The pictures displayed aimed at prompting the activation of the respective group stereotypes of warmth and competence, and participants were instructed to view each character as a representative member for the specific social group targeted. It can therefore be assumed that our participants considered the characters as a function of social group affiliations rather than as individual targets.

Participants also rated how warm and competent they perceived these characters to be (manipulation check; S3 Fig, panel A). The character in question was displayed in the center of the screen and participants could mark their choice on two continuous visual analog scales (presented in two consecutive windows), ranging from 0 (“not at all warm”/“not at all competent”) to 100 (“very warm”/“very competent”). Lastly, participants also completed the Inclusion of Other in the Self (IOS; [59]) scale, specifying the degree of perceived overlap between themselves and any of the four target characters (S3 Fig, panel B). The IOS scale consists of seven pairs of circles that vary in their degree of overlap—from 1 (very low overlap) to 7 (almost complete overlap). The participants’ task was to select, for each character, the pair of circles that best described their relationship with this character.

Characters and backgrounds representing the different scenarios were created with *The Sims 4* (Electronic Arts, CA, USA). The resulting 128 visual stimuli (each of the four characters displayed in each of the 32 scenarios) were controlled in brightness and contrast by using MATLAB R2017a (The Math Works, Inc., MA, USA). The experiment was programmed with E-Prime 2.0 Professional (version 2.0.10.353; Psychology Software Tools, Pittsburgh, PA, USA).

### Procedure

Participants were informed that the aim of the study was the development of new stimuli for upcoming studies. About half of the participants (n = 49 of 89 participants in total) were equipped with sensors that captured diverse physiological responses (e.g., electrocardiogram; data not presented in the present manuscript). After having signed a written informed consent form, they engaged in six experimental blocks that were presented in a fixed sequence. The first two blocks assessed (over)optimistic expectancies for the self and the different characters displayed. These data are unrelated to the present research aims and have been analyzed separately (see [60] for details). Consequently, they are not presented here. The fourth block included ratings of subjective controllability, valence, frequency in the general population, emotional impact, and personal experience for each of the 32 scenarios included (see S1 Table for details) and are also not further considered.

Notably, the third block comprised ratings of cognitive and emotional empathy for all scenario (n = 32) × character (n = 4) combinations (resulting in 128 ratings for each type of empathy; see previous section; S2 Fig). The fifth block consisted of the completion of the IOS scale (S3 Fig, panel B), with the aim of measuring social identification with the different characters (four ratings in total). Finally, the sixth block comprised ratings for each character on the dimensions warmth and competence (four ratings for each perceived warmth and perceived competence; manipulation check; S3 Fig, panel A). Before our participants left the laboratory, they were debriefed.

## Data preparation and analysis

### Data preparation

Cognitive and emotional empathy ratings for undesirable scenarios were reversed in order to reflect the general appropriateness of the attributed (cognitive empathy) or experienced (emotional empathy) feeling states (i.e., greater scores representing greater (assigned) suffering; we note that the response scales ranged from– 50 [feeling “very bad”] to 50 [feeling “very good”]). For the desirable scenarios, no recoding was needed because greater scores already reflected more positive feelings attributed to the depicted character (cognitive empathy) or felt by the participant (emotional empathy).

### Data analysis

First, we calculated Pearson product-moment correlation coefficients in order to investigate the distinctive character of the various constructs (cognitive and emotional empathy expressed, social identification with the characters, and perceived warmth and competence of the characters) included in our study. In addition to providing this information across all scenarios (i.e., entering averaged scores per participant across all scenarios considered), we included separate correlation coefficients for negative (i.e., undesirable) and positive (i.e., desirable) scenarios.

We used linear mixed modeling to address all of our research aims, as implemented in the GAMLj module in jamovi (The jamovi project (2020). jamovi. (Version 1.2) [Computer Software]. Retrieved from https://www.jamovi.org.). One important reason for using this statistic over traditional methods is that it allows us to account for both participant- and scenario-related variance in one model (see [61] for a more detailed account of the rationale for using this approach, and [62] for a recent implementation of this method for repeated measures designs). To elucidate any interaction effects, we used the simple effects procedure as implemented in jamovi/GAMLj.

Two separate linear mixed models were computed to investigate our three research aims—one for cognitive empathy and one for emotional empathy. Both models had a completely crossed design, with crossed random effects for participants (n = 89) and scenarios (n = 32), which were both at level 2, and with ratings of emotional and cognitive empathy, respectively, for each of the four characters within each combination of participant and scenario (level 1 data). The first linear mixed model had cognitive empathy as the level 1 outcome, whereas the second model had emotional empathy as the level 1 outcome. For both models, *perceived warmth* (0–100) and *perceived competence* (0–100), as well as *IOS* (1–7), were level 1 continuous predictors (centered on the mean). *Valence* (positive, negative) was a level 2 categorical predictor, as it represented a characteristic of scenarios.

The manipulation check, based on our participants’ warmth and competence ratings for the different characters, revealed, among other things, that the elderly character was not perceived as incompetent as intended (S1 Analysis); therefore, we decided to include the participants’ actual ratings of warmth and competence in our analyses rather than relying on factors. However, because warmth and competence have been used as categorical predictors in most previous studies, we additionally ran the models with *warmth* (levels: cold, warm) and *competence* (levels: incompetent, competent) as factors. In these models (with warmth and competence as categorical predictors), random slopes of *warmth* and *competence* were included for both subject variables (participants and scenarios) in addition to a random slope of *valence* for the subject variable participants (since valence is an attribute of scenarios, its effect could vary only across participants, not across scenarios). The results of these additional models are described in S2 Analysis.

For the main analyses with *perceived warmth* and *perceived competence* as continuous level 1 predictors, including random slopes of these main effects prevented convergence, which led to the decision of keeping only the participants’ random slope of *valence* for these models. All models were estimated with an unstructured covariance matrix (correlated random effects). The included random effect parameters were tested for significance by using likelihood ratio (LR) test comparisons with models that did not contain the respective effect in question. For both dependent variables, we started with an intercept-only model to estimate the intra-class correlation coefficients (ICCs) related to the random intercepts of participants and scenarios. In the next step, models with *(perceived) warmth*, *(perceived) competence*, *valence*, and *IOS* as main (fixed) effects, as well as their two-way, three-way, and four-way interactions, were specified. To avoid over-parametrization and unnecessary (and therefore potentially biasing) partialling of irrelevant higher order effects, we then removed all nonsignificant four- and three-way interactions to arrive at the final model for the respective dependent variable (cognitive empathy and emotional empathy; for details, see the respective analyses below). To obtain effect sizes for the fixed effects, we additionally computed the models with grand-mean standardized dependent variables, as well as continuous predictors [63,64]. The resulting standardized effect estimates *b*_*s*_ represent the effects in the standard deviation metric and can therefore be interpreted as independent of the scaling/variances of independent and dependent variables. In the case of a dichotomous categorical predictor, the predictor was not standardized, as this would obscure the interpretation of the effects rather than elucidate it. The resulting partially standardized effect represents a standardized mean difference between the groups that the predictor represents and thus can be interpreted as being similar to Cohen’s *d*. Both fully standardized effect estimates of continuous predictors and partially standardized effect estimates of dichotomous predictors are referred to as *b*_*s*_ below to avoid unnecessary complexity in statistical notation.

## Results

### Correlations between the included variables

Table 1 and S2 Table (the latter providing separate correlations for the different targets and valence levels) show the patterns of Pearson product-moment correlations between the variables included in our analyses. Unsurprisingly, our data demonstrate an overlap between the two empathy measures. Yet, there was only about 30% of shared variance between the two constructs (which matches the earlier observed commonality between empathic concern and perspective taking across different populations; cf. [65]). Therefore, it was justified to perform separate analyses for cognitive and emotional empathy.

**Table 1:** Pattern of correlations between the different constructs.

		Cognitive Empathy	Emotional Empathy	Social Identification	Perceived Warmth
Emotional Empathy	r	.575***			
	p	< .001			
Social Identification	r	.109	.196		
	p	.309	.066		
Perceived Warmth	r	.126	.293**	.201	
	p	.239	.005	.059	
Perceived Competence	r	.228*	.344***	.317**	.579***
	p	.032	< .001	.002	< .001

Note.

*p < .05,

**p < .01,

***p < .001,

*n* = 89 participants. Correlations are collapsed across all levels of target character and scenario valence.

### Cognitive empathy (mentalizing, attribution of feelings)

The intercept-only model for cognitive empathy yielded ICCs of 0.134 and 0.239 for participants and scenarios, respectively, indicating that 13.4% of overall variance occurred among participants and 23.9% among scenarios. The respective LR tests for the random intercept variances for participants and scenarios were both highly significant, with LR *χ*^2^(1) = 1884.930, *p* < .001, and LR *χ*^2^(1) = 3419.969, *p* < .001, respectively. With regard to information criteria, the Akaike information criterion (AIC) and Bayesian information criterion (BIC) of the intercept-only model were 91215.232 and 91241.881, respectively. For the model including all predictors (perceived warmth, perceived competence, valence, IOS), as well as all possible (two-way, three-way, and four-way) interactions as fixed effects, AIC and BIC were 90247.508 and 90532.232, respectively. In this model, the four-way interaction (*p* = .730) and all four three-way interactions were nonsignificant (perceived warmth × valence × IOS: *p* = .880; perceived competence × valence × IOS: *p* = .062; perceived warmth × perceived competence × IOS: *p* = .518; perceived warmth × perceived competence × valence: *p* = .608). After we removed the nonsignificant four-way and three-way interactions, the information criteria (AIC = 90242.322, BIC = 90427.630) of the final linear mixed model (including main effects and all two-way interactions) were both lower than for the full model above, and the model showed a significant random slope variance of valence across participants (LR *χ*^2^(2) = 275.771, *p* < .001).

**Fig 1:**
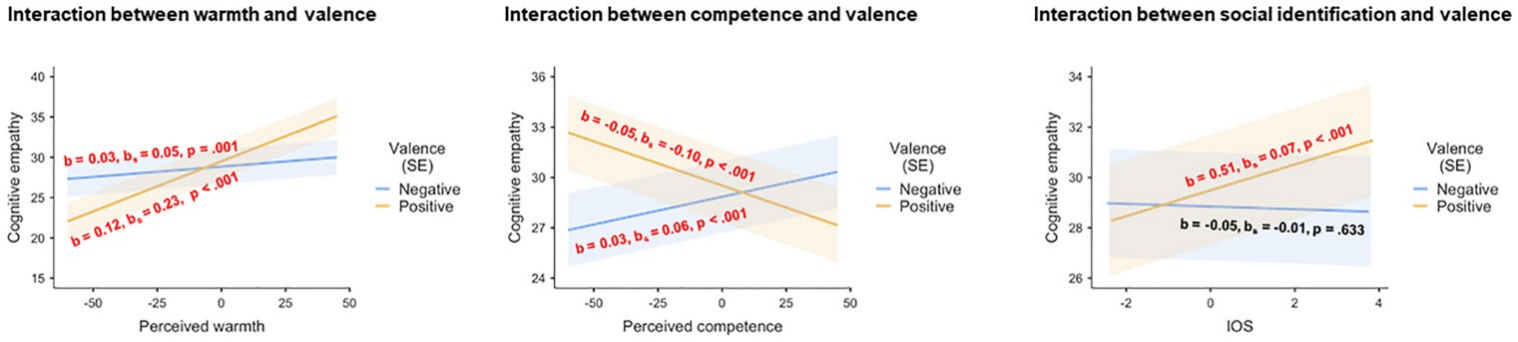
Simple effects of perceived warmth, perceived competence, and social identification on cognitive empathy for negative and positive scenarios. Left panel: Warmth ratings and scenario valence; middle panel: Competence ratings and scenario valence; right panel: Social identification and scenario valence. IOS = Inclusion of Other in the Self scale, measuring the extent of social identification. SE = standard error.

We predicted (a) cognitive empathy to be reduced for targets perceived as low in both warmth and competence (i.e., interactive effects of attributed warmth and competence). Yet, we did not observe a statistically significant interaction between perceived warmth and perceived competence (*F*(1, 11256.2) = 1.61, *p* = .205) in the current study. Consistent with our hypothesis of (b) greater effects of the perceptual dimensions in positive than in negative scenarios, however, the final model revealed a main effect of perceived warmth (*F*(1, 11262.8) = 147.27, *p* < .001) that was qualified by an interaction of perceived warmth with valence (*F*(1, 11039.5) = 98.60, *p* < .001). Simple effects analyses revealed that respondents reported higher cognitive empathy for warm characters than for cold characters—particularly for the positive scenarios (positive; *b* = 0.124, *b*_*s*_ = 0.232; *t*(11296.3) = 15.66, *p* < .001; negative: *b* = 0.026, *b*_*s*_ = 0.048; *t*(11233.7) = 3.22, *p* = .001) (see Fig 1, left panel). Furthermore, there was an interaction of perceived competence with valence (*F*(1, 11259.6) = 116.32, *p* < .001): Cognitive empathy varied as a negative function of perceived competence in the positive scenarios (*b* = -0.053, *b*_*s*_ = -0.101; *t*(11279.9) = -7.82, *p* < .001), but, somewhat surprisingly, as a positive function of perceived competence in the negative scenarios (*b* = 0.033, *b*_*s*_ = 0.063; *t*(11253.48) = 4.92, *p* < .001) (see Fig 1, middle panel).

In addition, we predicted (c) that cognitive empathy would vary as a positive function of social identification, and we explored possible interactions between social identification and the other variables in the prediction of cognitive empathy. In accordance with our hypothesis, we observed a main effect of the degree of social identification with the target character (*F*(1, 11257.9) = 6.87, *p* = .009), which was qualified by an interaction of degree of identification with valence (*F*(1, 10389.8) = 15.64, *p* < .001). Simple effects showed that cognitive empathy increased as a function of social identification with the target character during positive scenarios (*b* = 0.514, *b*_*s*_ = 0.067; *t*(11239.8) = 4.53, *p* < .001), whereas social identification did not matter for negative scenarios (*b* = -0.054, *b*_*s*_ = -0.007; *t*(11024.1) = -0.48, p = .633) (see Fig 1, right panel).

**Fig 2:**
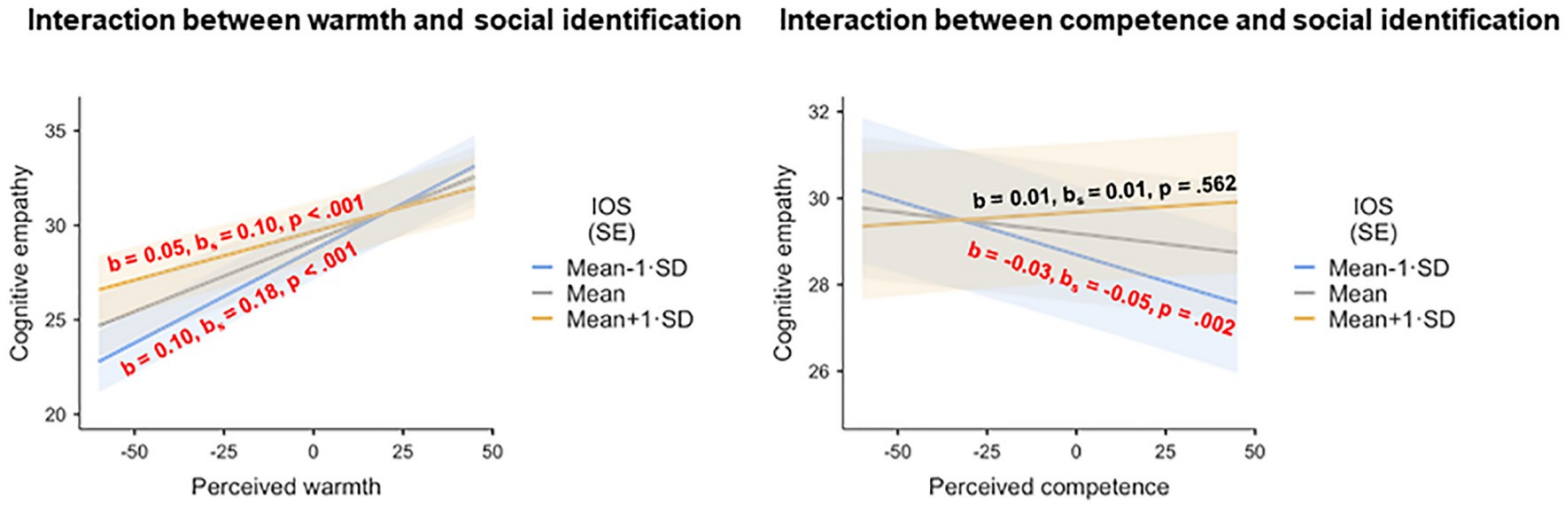
Simple effects of perceived warmth and perceived competence on cognitive empathy at different levels of social identification. Left panel: Warmth ratings and social identification; right panel: Competence ratings and social identification. IOS = Inclusion of Other in the Self scale, measuring the extent of social identification; SE = standard error.

Furthermore, we found an interaction of degree of identification with perceived warmth (*F*(1, 11236.2) = 11.71, *p* < .001). Although all three simple slopes were significant, their positivity decreased with increasing social identification, indicating greater influence of perceived warmth at low levels than at high levels of social identification (social identification = Mean– 1 SD: *b* = 0.099, *b*_*s*_ = 0.183; *t*(11262.3) = 14.49, *p* < .001; social identification = Mean: *b* = 0.075, *b*_*s*_ = 0.140; *t*(11262.8) = 12.14, *p* < .001; social identification = Mean + 1 SD: *b* = 0.051, *b*_*s*_ = 0.096; *t*(11249.4) = 4.61, *p* < .001) (see Fig 2, left panel).

A further significant interaction was observed for social identification and perceived competence (*F*(1, 11262.8) = 4.95, *p* = .026). Simple effects analyses showed that when participants barely identified with a target character, increasing perceptions of competence in a target character were associated with decreasing cognitive empathy ratings (social identification = Mean– 1 SD: *b* = -0.025, *b*_*s*_ = -0.048; *t*(11262.8) = -3.04, p = .002). Notably, this association between perceived competence and cognitive empathy ratings shrank with increasing levels of social identification and was no longer significant for medium and high levels of social identification (social identification = Mean: *b* = -0.010, *b*_*s*_ = -0.019; *t*(11251.0) = -1.80, *p* = .072; social identification = Mean + 1 SD: *b* = 0.005, *b*_*s*_ = 0.010; *t*(11258.0) = 0.58, *p* = .562) (see Fig 2, right panel). There was no main effect of valence (*F*(1, 31.9) = 0.05, *p* = .822) or competence (*F*(1, 11251.0) = 3.25, *p* = .071).

### Emotional empathy (affective sharing, own feelings)

The intercept-only model for emotional empathy revealed ICCs of 0.213 and 0.135 for participants and scenarios, respectively, indicating that 21.3% of overall variance occurred among participants and 13.5% among scenarios. Random intercept variances for participants and scenarios were both highly significant, with LR *χ*^2^(1) = 2848.503, *p* < .001, and LR *χ*^2^(1) = 1939.140, *p* < .001, respectively. The AIC and BIC of the intercept-only model were 89855.762 and 89882.872, respectively. For the model that included all predictors (perceived warmth, perceived competence, valence, IOS) and all possible (two-way, three-way, and four-way) interactions as fixed effects, the AIC and BIC were 88381.350 and 88669.491, respectively. In this model, the four-way interaction (*p* = .334) and three of the four three-way interactions were nonsignificant (perceived warmth × valence × IOS: *p* = .457; perceived competence × valence × IOS: *p* = .618; perceived warmth × perceived competence × valence: *p* = .065). After we removed the nonsignificant four-way and three-way interactions, the information criteria (AIC = 88381.144, BIC = 88593.167) of the final linear mixed model (including main effects, all two-way interactions, and the three-way interaction perceived warmth × perceived competence × IOS) were both lower than for the full model, and the model showed a significant random slope variance of valence across participants (LR *χ*^2^(2) = 400.362, *p* < .001).

**Fig 3:**
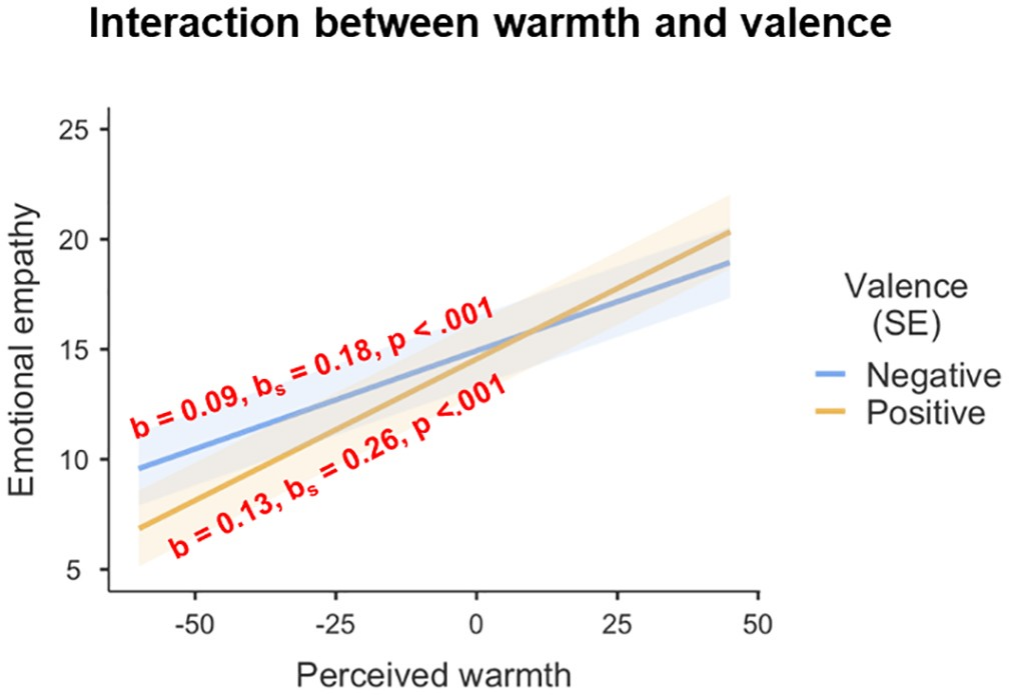
Simple effects of perceived warmth on emotional empathy for negative and positive scenarios. SE = standard error.

We expected emotional empathy to vary (a) as a positive function of perceived warmth and (b) as a negative function of perceived competence. Moreover, we predicted (c) greater effects of the perceptual dimensions in positive than in negative scenarios. In line with these hypotheses, the final model revealed a main effect of perceived warmth (*F*(1, 11252.8) = 334.82, *p* < .001), qualified by an interaction of perceived warmth with valence (*F*(1, 11169.7) = 18.29, *p* < .001; Fig 3). Simple effects showed that respondents reported higher emotional empathy for warm characters than for cold characters in both types of scenarios, but more so in the positive scenarios (positive: *b* = 0.128, *b*_*s*_ = 0.259; *t*(11287.8) = 17.09, *p* < .001; negative: *b* = 0.089, *b*_*s*_ = 0.180; *t*(11275.9) = 11.88, *p* < .001). The main effect of perceived warmth was also qualified by interactions with perceived competence and with levels of social identification (see below).

Consistent with our prediction of a negative link between attributions of competence and emotional empathy, a significant main effect of perceived competence (*F*(1, 11260.0) = 32.02, *p* < .001) indicated that overall, higher competence levels were related to lower emotional empathy (*b* = -0.034, *b*_*s*_ = -0.070), but this effect was qualified by interactions with several other factors. Specifically, our analyses further yielded significant interactions between perceived warmth and perceived competence (*F*(1, 11259.6) = 11.34, *p* < .001), between perceived warmth and social identification (*F*(1, 11262.0) = 11.71, *p* < .001), and between perceived competence and social identification (*F*(1, 11249.4) = 5.84, *p* = .016).

**Fig 4:**
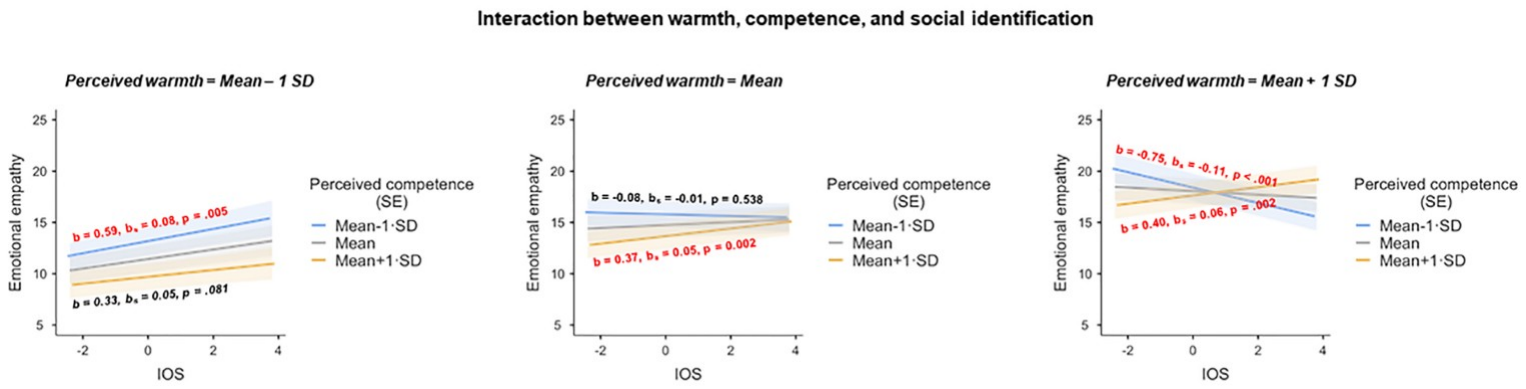
Interaction effects between social identification, warmth, and competence on ratings of emotional empathy. SE = standard error.

Notably, those effects were in turn qualified by a three-way interaction of social identification with perceived warmth and perceived competence (*F*(1, 11261.7) = 16.25, *p* < .001; Fig 4), which relates to our hypothesis that (d) cognitive empathy varies as a positive function of social identification (including the exploration of possible interactions between social identification and the other variables in the prediction of emotional empathy). For cold characters (perceived warmth = Mean– 1 SD), emotional empathy increased as a function of social identification, and this was relatively independent of level of perceived competence (perceived competence = Mean– 1 SD: *b* = 0.594, *b*_*s*_ = 0.084; *t*(11261.8) = 2.79, *p* = .005; perceived competence = Mean: *b* = 0.463, *b*_*s*_ = 0.065; *t*(11261.8) = 3.07, *p* = .002; perceived competence = Mean + 1 SD: *b* = 0.331, *b*_*s*_ = 0.047; *t*(11258.1) = 1.74, *p* = .081). With rising levels of perceived warmth, effects of social identification became more dependent on perceived competence and thus more interactive. For warm characters (perceived warmth = Mean + 1 SD), emotional empathy increased as a function of social identification only if the character was perceived as competent (perceived competence = Mean + 1 SD: *b* = 0.404, *b*_*s*_ = 0.057; *t*(11261.5) = 3.08, *p* = .002) and decreased as a function of social identification if the character was perceived as having average competence (perceived competence = Mean: *b* = -0.173, *b*_*s*_ = -0.024; *t*(11256.5) = -1.98, *p* = .047) or being incompetent (perceived competence = Mean– 1 SD: *b* = -0.751, *b*_*s*_ = -0.106; *t*(11257.6) = -4.57, *p* < .001) (see Fig 4). There were no significant main effects of valence (*F*(1, 34.6) = 0.03, *p* = .862) or social identification (*F*(1, 11258.4) = 3.20, *p* = .074), nor were there statistically significant interaction effects between valence and social identification (*F*(1, 10806.5) = 2.06, *p* = .151) or between valence and perceived competence (*F*(1, 11261.3) = 0.29, p = .593).

## Discussion

Only a few studies [39,40,46,47] have investigated emotional empathic responding for varying perceived target dimensions as defined by the SCM (i.e., warmth and competence). Although some findings [45] are suggestive of the hypothesis that emotional empathy is more strongly influenced by the dimension of warmth than by the dimension of competence, others point to the possibility of combined effects of warmth and competence (e.g., [39,40]). Even less is known about cognitive empathy. Given the assumption that cognitive and emotional empathy are separate but interrelated concepts, an open issue is how strongly the findings for cognitive and emotional empathy resemble each other. This question remains to be answered because no single study, to our knowledge, has examined both types of empathy with the same experimental paradigm [14]. Moreover, it is unknown as to how the valence of an imagined scenario, on the one hand, and social identification with the social targets, on the other, might moderate the influence of warmth and competence perceptions on cognitive and emotional empathy. The current study was performed to address these questions.

The following set of hypotheses was established: We expected (a) cognitive empathy to reveal interactive effects of attributed warmth and competence (i.e., to be reduced in social targets perceived as cold and incompetent); (b-c) emotional empathy to vary as a positive function of perceived warmth and as a negative function of perceived competence (resulting in increased emotional empathy for targets perceived as high in warmth but low in competence, and decreased emotional empathy for targets perceived as low in warmth but high in competence). Furthermore, we predicted (d) a stronger influence of attributed warmth and competence for desirable compared with undesirable events (for both types of empathy) and (e) increases in cognitive and emotional empathy associated with increasing levels of social identification. In addition, we explored the possibility of interactions of social identification with the two perceptual dimensions and scenario valence. Table 2 displays a summary of our findings in relation to these hypotheses.

**Table 2:** Summary of results.

Hypotheses	Supported by available data?
*Cognitive Empathy*	
- Interactive effects of perceived warmth and competence	no
- Greater reliance on perceptual dimensions in positive than in negative scenarios	yes (perceived warmth), no (perceived competence)
- Positive influence of social identification	yes (positive scenarios), no (negative scenarios)
*Emotional Empathy*	
- Positive influence of perceived warmth	yes
- Negative influence of perceived competence	yes (except for warm others that the participants strongly identified with)
- Greater reliance on perceptual dimensions in positive than in negative scenarios	yes (perceived warmth), no (perceived competence)
- Positive influence of social identification	yes (except for warm incompetent others)

### Cognitive empathy (mentalizing, attribution of feelings)

From the existent (preliminary) evidence in the field of perspective taking [46,47,66], we predicted interactive effects of attributed warmth and competence to result in reduced cognitive empathy for individuals perceived as cold and incompetent (here represented by the alcoholic character). We did not replicate this finding, however. In the analyses performed, none of the interactions comprising warmth and competence turned out to be significant. Furthermore, there were no main effects of perceived warmth and perceived competence. Instead, effects of these perceptual dimensions were dependent on both the valence of a scenario and the level of identification with the social targets. Specifically, we observed the positive association between participants’ warmth ratings and their specified cognitive empathy to be somewhat larger for positive than for negative scenarios (note, however, that the association was significant in both cases), which accords with the observation that people more commonly engage in the search for explanations regarding bad outcomes than they do regarding good outcomes [51–53]. Our results are thus in line with our hypothesis that empathic responding to positive outcomes relies more strongly on the stereotypical properties of the social targets in question, thereby bypassing time-consuming, effortful searches for explanations or justifications.

Notably, however, the existing significant interaction between perceived competence and valence is not unequivocally consistent with such an interpretation. Whereas for positive scenarios higher cognitive empathy was expressed for low than for high competent social targets, the reverse was true for negative scenarios. Therefore, our valence-specific processing hypothesis regarding the impact of perceptual properties needs further investigation. One possibility (among others) is that it holds for perceptions of warmth but not for perceptions of competence. Alternatively, the pattern arising for competence may possibly be explained by reference to the specificities of the social characters included in our study. In general, the low competent social targets (here represented by the alcoholic character and—to a lesser extent—the elderly character; see S1 Analysis) should have been perceived as being in a worse overall state than the high competent social targets (here represented by the student and the businessperson). Therefore, the incompetent targets may have been assumed to not experience particularly strong negative emotions if something undesirable (i.e., usual) happened, but instead to have strong positive emotions if something desirable (i.e., unusual) happened (with the opposite effect holding for the competent targets).

In line with our hypothesis, the degree of social identification significantly predicted cognitive empathy, such that a higher level of identification with the characters was associated with increased cognitive empathy. Yet, the significant interaction between IOS scores and scenario valence revealed that this association held only for positive scenarios, and cognitive empathy did not vary as a function of social identification for negative scenarios. This observation could again point to a greater reliance on shortcut processing when stating cognitive empathy for desirable rather than undesirable outcomes. Consistent with such an interpretation, society tends to impose stricter rules on how to empathize in negative situations (everybody has the right to feel bad), thereby not leaving much space for variations in cognitive empathy. By contrast, there are no such rules for positive situations, hence letting people freely engage in the attribution of stronger positive feelings to those who they more intimately identify with.

Moreover, the level of social identification interacted with both SCM perceptual dimensions (interactions between IOS and perceived warmth and between IOS and perceived competence). Specifically, although cognitive empathy for the warm characters was virtually unaffected by the degree of social identification, cognitive empathy for the cold characters increased as the degree of social identification also increased. Thus, increasing levels of warmth reduced the positive effects of social identification on cognitive empathy. We attribute this to a potential ceiling effect of the participants’ identification with the warm characters. Similarly, increasing levels of social identification reduced the positive effects of warmth perceptions on cognitive empathy, which speaks to a reduced reliance on perceptual properties attributed to a social target in the case of strong social identification. The positive interaction between social identification and perceived competence further supports such an interpretation of our data because increasing levels of social identification additionally reduced the negative effect exerted by increasing levels of competence.

### Emotional empathy (affective sharing, own feelings)

Earlier research on the SCM suggested a positive association between perceived warmth and displayed empathy (e.g., [67]). Furthermore, emotional empathy has been reported to be inversely related to the level of competence attributed to a social target [39–42]. Thus, we predicted increasing levels of warmth to yield positive changes in emotional empathy and increasing levels of competence to yield negative changes. Furthermore, these effects were thought to be stronger for desirable than for undesirable outcomes (the latter being supposedly characterized by greater attempts at searching for explanations).

Results in the present study are mostly consistent with these hypotheses. First, we indeed found convincing evidence for an influence of perceived warmth on emotional empathy that was moderated by the valence of the scenarios. As expected, characters perceived as warm evoked greater emotional empathy than did characters perceived as cold, and this was stronger for positive than negative outcomes. This finding aligns well with the observation that we generally hold more positive attitudes for warm characters and display more favorable behaviors toward them [9,33].

We further observed a significant main effect of perceived competence on emotional empathy: Attributions of incompetence evoked greater emotional empathy than did attributions of competence. Consistent with our predictions, the combination of these effects reveals that the lowest emotional empathy was expressed for cold and competent (supposedly envied; represented by the businessperson) targets. Thus, sharing emotions with envied social groups or individuals may be particularly difficult [67,68]. Positive feelings arising from seeing others experience desirable outcomes may be especially troublesome for those social targets that are perceived as already owning excessive parts of the available resources. Similarly, there is no reason why people should feel very bad about a misfortune happening to a person who they usually begrudge.

Our expectations of the highest emotional empathy being displayed toward the warm-incompetent subgroup (i.e., the elderly character), however, was not unequivocally supported (which may be related to the fact that the elderly character did not adequately map into its intended SCM quadrant; see S1 Analysis). In lieu of this, stereotypes of warmth and competence interacted with the degree of social identification to determine the levels of emotional empathic responses. This finding thus underscores that—instead of solely focusing on perceived warmth and competence—it is in further essential to consider the extent of identification with a social target. Respondents reported higher emotional empathy for the cold characters the more strongly they identified with these characters–independent of the level of competence displayed (Fig 4, left panel, shows in addition that—consistent with our interpretation of the data in the previous paragraph—the cold-competent subgroup was characterized by the lowest emotional empathy at each level of social identification). By contrast, for warm characters, emotional empathy increased as a function of social identification only if the target was perceived as competent, but unexpectedly decreased as a function of social identification if the target was perceived as incompetent. In sum, therefore, social identification with another person generally increased emotional empathy (which aligns with our hypothesis regarding the impact of social identification on emotional empathy), except for those targets that had been perceived as warm and incompetent. It remains to be clarified why the effect is reversed in their case.

### Comparison of effects observed for cognitive and emotional empathy

Results for cognitive and affective empathy were only partly overlapping, justifying our separate analyses for these constructs. Although it was true that warmth effects were similarly moderated by scenario valence in the prediction of both types of empathy, there were two noteworthy differences. First, scenario valence mattered more for cognitive than for emotional empathy because it additionally moderated the effects of perceived competence and social identification in the former case. Second, for cognitive empathy, increasing levels of social identification boosted the cognitive empathy reported and reduced the positive effects exerted by (a) elevated levels of warmth and (b) reduced levels of competence, with no interaction between warmth and competence ratings. By contrast, for emotional empathy, social identification interacted with both perceived warmth and perceived competence: Stronger identification with a social target resulted in increased emotional empathy expressed, except for warm-incompetent targets. These observed differences need further investigation.

## Limitations

Assessment of empathy in the current studies might be criticized. First, from earlier findings in the literature [40], we decided not to ask people separately how bad or how good they felt (or thought that the social target felt, for cognitive empathy) about imagining the different social targets in the depicted scenarios. However, results might have been different if we had opted to assess those two questions separately for both cognitive and emotional empathy. For instance, there might be value in assessing ambivalent emotional reactions to stimuli that can only be measured by assessing positive and negative feelings on separate scales [69]. Furthermore, earlier research on undesirable situations suggests that positive affective responses arising in an observer when seeing the misfortune of another relate to dispositional cognitive empathy, whereas negative affective responses relate to dispositional emotional empathy [16]. Although our study assessed cognitive and emotional empathy as situationally based (instead of targeting dispositions), these considerations warrant further inspection. Therefore, in future investigations, may want to separate positive and negative affective responses for both undesirable and desirable scenarios.

Second, it might be questioned whether high scores on the scale included in the current study adequately address cognitive empathy for the different characters. It is imaginable that those participants who denote that the social target is feeling particularly bad when facing a misfortune might exaggerate or overestimate those feelings, thereby demonstrating reduced rather than increased mentalizing capacities. However, this criticism must be addressed in the larger context of the entire research field and is not specific to the current study (notably, the methods that we used here are consistent with those of earlier research in the area; e.g., [39,40]).

Finally, the existence of gender differences in empathic responding [70,71] points to the possibility of quantitatively or qualitatively divergent determinants of cognitive and emotional empathy between men and women. Given that about 90% of our participants were female, the generalization of the present findings to both genders is questionable. Similar studies need to be conducted with (predominantly) male samples.

## Future directions

In future investigations in the field, researchers may consider the inclusion of psychophysiological measures such as facial electromyography or cardiac responding because they might reveal important insights that cannot be obtained with self-reported empathy (for evidence, see [40]). In fact, subjective ratings have been reported to be particularly prone to social desirability effects (for an overview of problematic issues related to self-reports, see [72]; for potential solutions, see [73,74]), which might bias research findings considerably. However, our findings for cognitive and emotional empathy are mostly in line with previous neuroimaging research and theoretical frameworks, thus increasing our trust in the reliability of the methodology used.

It would also be interesting to examine whether individualizing descriptions of the social target characters included in the current study—in particular descriptions regarding the targets’ mental states—change the pattern of responses observed. Recent findings in the field imply that such descriptions can decrease parochial empathy (i.e., intergroup empathy bias representing greater empathy expressed for in-groups [32,75]).

Subsequent studies may further examine similarities and differences between affective and cognitive empathy for the different social targets, on the one hand, and feelings expressed for the self, on the other. Such a distinction would thus extend the differentiation between “imagining self” and “imagining other” made by other authors in studies on empathy and perspective taking [76,77] into “imagining self” vs. “imagining other—cognitive empathy” vs. “imagining other—emotional empathy.”

Finally, predominant approaches in the literature (e.g., [8,45]) claim a primacy of perceptions of warmth (i.e., communal content) over competence (i.e., agentic content) in social interactions. Recent developments in the area (e.g., [8]) further suggest that the weight given to each content depends on the kind of perspective taken by an individual. Specifically, stronger ponderation of warmth (than of competence) should arise if one adopts an observer or recipient perspective in social interactions, with the reverse holding true for the adoption of an actor perspective. In the current study, one might be tempted to assume that participants adopted an observer perspective (the focus being on the social target and not on the self, combined with the target “receiving” desirable or “undesirable” outcomes). If this were true, it would imply that our results do not support the idea of such differences being transferrable to the empathy context. Yet, we did not assess the type of perspective taken by our participants. In subsequent studies, researchers may therefore consider systematic manipulations of perspective taking.

## Summary and conclusions

Accounts of empathy distinguish between cognitive (attribution of mental states to others) and emotional (sharing of emotions with a social target) empathy and have led to numerous investigations in the field. Advantages of the current study over earlier investigations relate to (a) the simultaneous assessment of cognitive and affective empathy (i.e., with the same experimental paradigm; [14]) for various social targets that can be classified along two universal dimensions of social perception (i.e. warmth and competence); (b) the consideration of both positive and negative scenarios; and (c) the additional examination of social identification.

Perceived warmth and competence predicted empathic responses in positive situations but a little less in negative situations, which may be explained by greater personal liberty of response to positive (rather than to negative) social outcomes. Although society exerts general pressure on people to display regret toward others who suffer misfortunes (instead of showing malicious joy), it does not require the display of joy toward other people’s fortunes. Our results thus clearly demonstrate the importance of considering the desirability of the situation when investigating effects related to cognitive and emotional empathy.

Moreover, our findings related to social identification open the possibility of promoting empathic responses—especially toward cold social targets—by manipulating the degree of social identification. Social identification can be boosted in multiple ways. The most direct and intuitive method is to establish more common ground (e.g., beliefs, values) between oneself and another person. However, this might not always be feasible. An alternative method consists of increasing the comparative fitness between the self and target other by determining a common “antagonistic” environment [78,79]. For example, social campaigns against the marginalization of groups perceived as unfriendly, untrustworthy, or unpredictable (e.g., immigrants or those struggling with mental illness) could adapt their message by emphasizing that both the audience of the campaign and the marginalized individuals share the same daily hassles or basic needs (i.e., the same environment).

## Supporting information

S1 Fig Visualization of included social targets. (PNG)

S2 Fig Visualization of empathy ratings. (PNG)

S3 Fig Visualization of warmth, competence, and social identification ratings. (PNG)

S1 Table Exhaustive list of events used in the experiment. (DOCX)

S2 Table Pattern of correlations between the different constructs. (DOCX)

S1 Analysis Ratings of social identification and perceived warmth and competence (manipulation check). (DOCX)

S2 Analysis Linear mixed models with warmth and competence as categorical predictors. (DOCX)

S1 Data Raw data of all described analyses. (XLSX)
