# Supplementary figures and images for: Empathic responses to social targets: The influence of warmth and competence perceptions, situational valence, and social identification
Source: PLoS One. 2021 Mar 15;16(3):e0248562. doi: 10.1371/journal.pone.0248562 (PMC7959363; doi:10.1371/journal.pone.0248562)

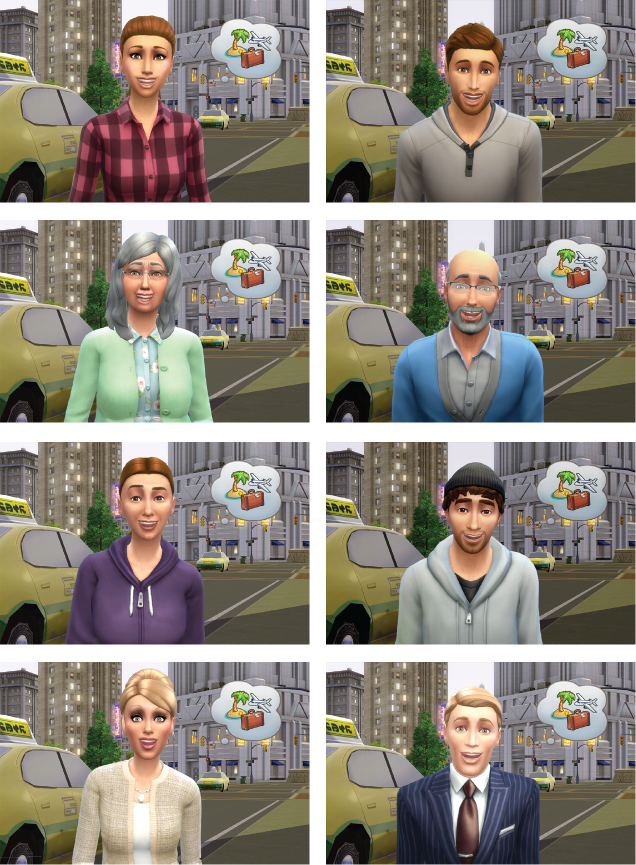

Supplement: S1 Fig — (PNG) [file pone.0248562.s001.png]

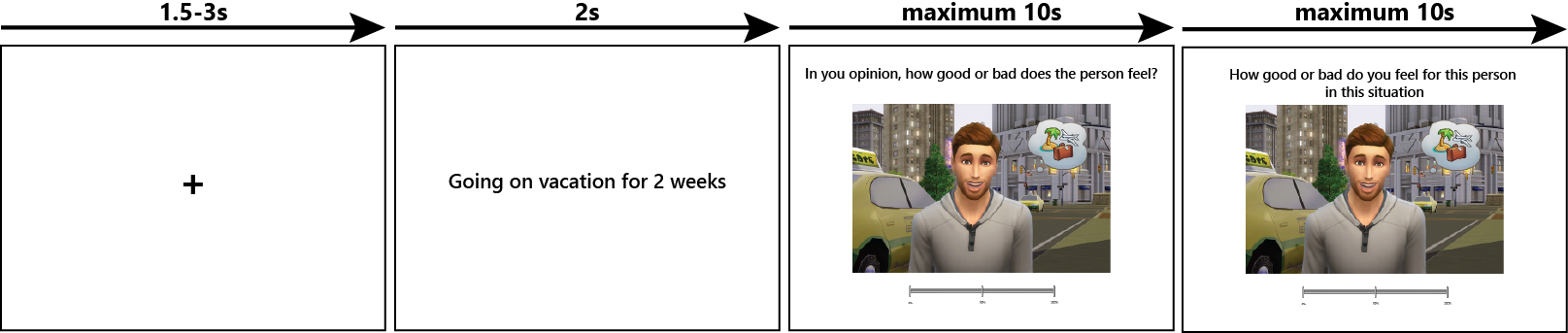

Supplement: S2 Fig — (PNG) [file pone.0248562.s002.png]

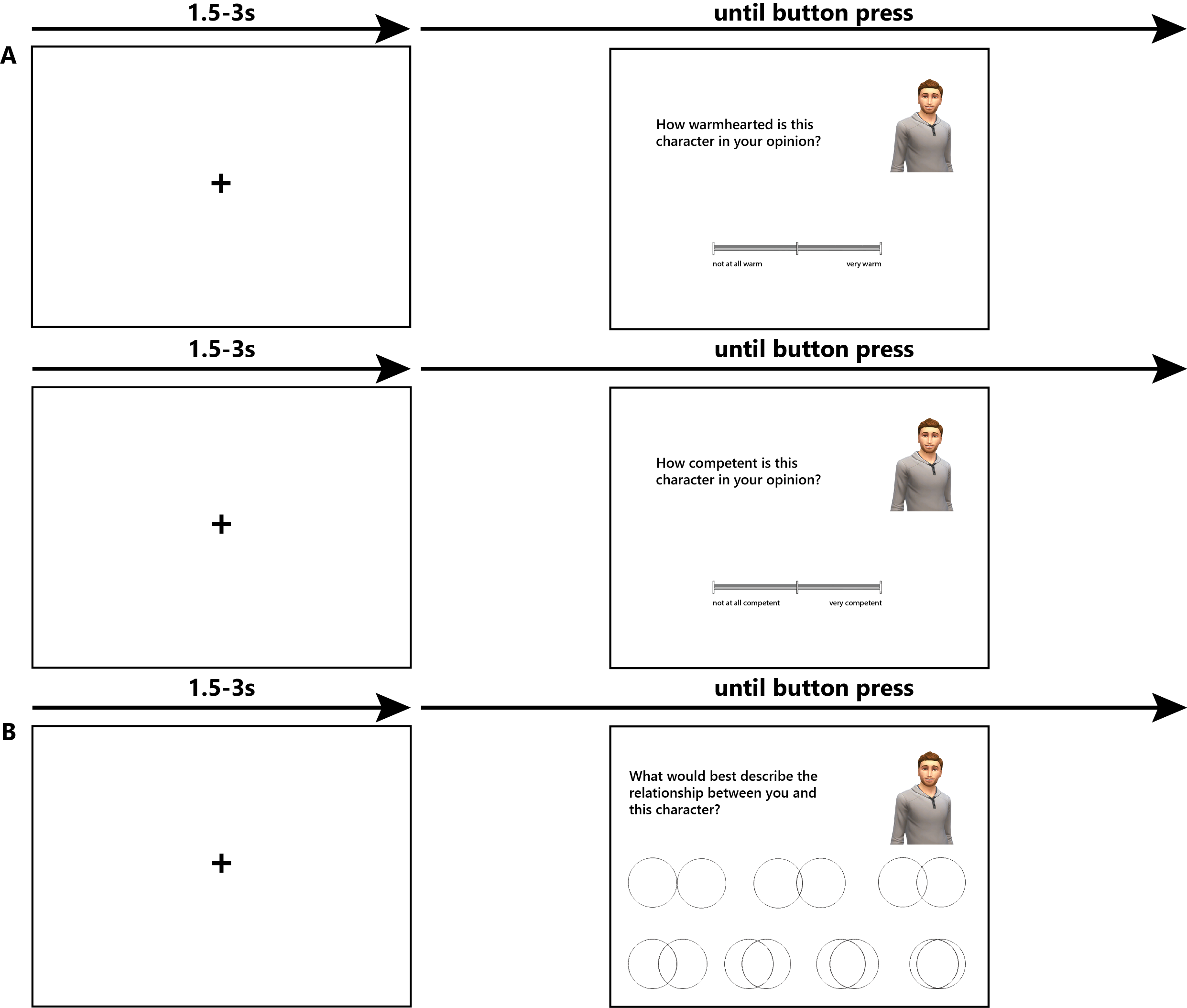

Supplement: S3 Fig — (PNG) [file pone.0248562.s003.png]
