# Supplementary material for: Empathic responses to social targets: The influence of warmth and competence perceptions, situational valence, and social identification
Source: PLoS One. 2021 Mar 15;16(3):e0248562. doi: 10.1371/journal.pone.0248562 (PMC7959363; doi:10.1371/journal.pone.0248562)
Supplement: S1 Table — (DOCX) [file pone.0248562.s004.docx]

**S1 Table. Exhaustive list of events used in the experiment.**

| Event | Desirability | Frequency | Controllability | Impact | Experience |
| --- | --- | --- | --- | --- | --- |
| Being hugged | 87.73 | 78.11 | 66.11 | 79.00 | 90.89 |
| A friend returns borrowed money to you | 72.39 | 58.82 | 57.93 | 43.76 | 65.46 |
| Being greeted warmly by relatives at a family celebration | 82.18 | 61.34 | 59.87 | 72.63 | 83.63 |
| Enjoy a warm bath or shower on a cold day | 85.74 | 67.52 | 92.27 | 50.77 | 81.83 |
| Hearing a very funny joke | 77.66 | 66.11 | 42.07 | 57.17 | 76.88 |
| New neighbor comes over to introduce themselves | 72.13 | 38.34 | 27.18 | 52.83 | 39.05 |
| A song that you like comes on the radio | 77.43 | 66.22 | 36.80 | 57.73 | 74.57 |
| Bumping into an old friend on the street | 79.20 | 52.29 | 18.51 | 68.40 | 64.37 |
| Delivering a speech successfully | 79.79 | 43.46 | 82.06 | 69.43 | 53.72 |
| A child has fun while you take care of him for 2 hours | 79.54 | 58.50 | 79.12 | 66.40 | 64.52 |
| Winning a karaoke contest | 75.13 | 24.32 | 60.52 | 63.04 | 12.66 |
| Writing a bestseller about one’s own life | 80.95 | 12.50 | 73.63 | 79.54 | 1.90 |
| Win a car in the lottery | 86.56 | 6.04 | 11.04 | 76.87 | 3.55 |
| Find a 20 CHF bill on the ground | 79.02 | 21.11 | 9.80 | 53.73 | 34.54 |
| Seeing a comet in the sky | 79.21 | 26.74 | 20.35 | 62.94 | 50.65 |
| Win a sports bet | 73.51 | 23.26 | 21.68 | 67.34 | 8.26 |
| The store closes just as you arrive | 30.83 | 51.17 | 79.34 | 45.93 | 51.67 |
| Finding rotten food in the refrigerator | 25.55 | 55.95 | 87.56 | 36.27 | 55.29 |
| Using a public restroom | 40.01 | 72.74 | 71.76 | 25.48 | 80.16 |
| Drinking cold coffee/tea | 40.28 | 62.79 | 84.83 | 26.32 | 62.66 |
| A neighbor is listening to very loud music | 33.27 | 53.07 | 30.20 | 50.00 | 47.10 |
| Getting heartbroken after a relationship | 6.62 | 48.12 | 34.24 | 97.48 | 51.50 |
| Being confused with another person | 46.07 | 44.22 | 13.06 | 37.11 | 48.49 |
| Computer crashes in the middle of writing a text | 14.73 | 45.61 | 31.23 | 68.15 | 54.78 |
| Marriage ends in a bitter divorce | 6.06 | 47.99 | 62.51 | 96.57 | 5.89 |
| Lose 50 CHF | 24.06 | 23.57 | 58.26 | 63.63 | 22.24 |
| Developing an excruciating toothache | 9.57 | 34.77 | 45.12 | 56.74 | 33.18 |
| Receiving a wound that needs to be sewn | 17.35 | 35.63 | 57.88 | 62.11 | 32.72 |
| Witnessing a bank robbery | 16.70 | 9.54 | 8.40 | 74.88 | 2.33 |
| Being falsely accused of a serious crime | 5.27 | 15.02 | 18.07 | 88.38 | 2.89 |
| Getting bitten by a dog | 12.18 | 23.59 | 34.23 | 67.39 | 19.29 |
| Witnessing a tree falling on a house | 16.23 | 9.50 | 7.32 | 68.34 | 5.33 |

*Note*. There are 32 positive and negative events with corresponding scores of desirability (valence), perceived frequency in the general population, perceived controllability, emotional impact, and personal experience with each event. Positive and negative events are balanced on all characteristics (except desirability). *n* = 89 participants.
