# Supplementary material for: Empathic responses to social targets: The influence of warmth and competence perceptions, situational valence, and social identification
Source: PLoS One. 2021 Mar 15;16(3):e0248562. doi: 10.1371/journal.pone.0248562 (PMC7959363; doi:10.1371/journal.pone.0248562)
Supplement: S2 Table — (DOCX) [file pone.0248562.s005.docx]

**S2 Table. Pattern of correlations between the different constructs: A. Across all scenarios, B. for positive scenarios, C. for negative scenarios.**

1. **Across all scenarios**

|  |  |  |  |  |  |  |  |  |  |  |
| --- | --- | --- | --- | --- | --- | --- | --- | --- | --- | --- |
| **Alcoholic person** |  |  | Cognitive Empathy |  | Emotional Empathy |  | Social Identification |  | Perceived Warmth | |
|  | Emotional Empathy | r | **.547** | *** |  |  |  |  |  |  |
|  |  | p | < .001 |  |  |  |  |  |  |  |
|  | Social Identification | r | .046 |  | .028 |  |  |  |  |  |
|  |  | p | .668 |  | .798 |  |  |  |  |  |
|  | Perceived Warmth | r | .139 |  | **.292** | ** | **.258** | * |  |  |
|  |  | p | .195 |  | .005 |  | .015 |  |  |  |
|  | Perceived Competence | r | -.052 |  | .208 |  | .109 |  | **.606** | *** |
|  |  | p | .630 |  | .050 |  | .310 |  | < .001 |  |
| **Businessperson** |  |  | Cognitive Empathy |  | Emotional Empathy |  | Social Identification |  | Perceived Warmth | |
|  | Emotional Empathy | r | **.558** | *** |  |  |  |  |  |  |
|  |  | p | < .001 |  |  |  |  |  |  |  |
|  | Social Identification | r | .164 |  | .211 | * |  |  |  |  |
|  |  | p | .124 |  | .047 |  |  |  |  |  |
|  | Perceived Warmth | r | .113 |  | **.351** | *** | **.271** | * |  |  |
|  |  | p | .290 |  | < .001 |  | .010 |  |  |  |
|  | Perceived Competence | r | .207 |  | .124 |  | .103 |  | -.120 |  |
|  |  | p | .052 |  | .249 |  | .335 |  | .265 |  |
| **Elderly person** |  |  | Cognitive Empathy |  | Emotional Empathy |  | Social Identification |  | Perceived Warmth | |
|  | Emotional Empathy | r | **.602** | *** |  |  |  |  |  |  |
|  |  | p | < .001 |  |  |  |  |  |  |  |
|  | Social Identification | r | .101 |  | .086 |  |  |  |  |  |
|  |  | p | .347 |  | .420 |  |  |  |  |  |
|  | Perceived Warmth | r | **.312** | ** | **.272** | ** | .138 |  |  |  |
|  |  | p | .003 |  | .010 |  | .196 |  |  |  |
|  | Perceived Competence | r | **.267** | * | **.317** | ** | .206 |  | **.310** | ** |
|  |  | p | .011 |  | .002 |  | .053 |  | .003 |  |
| **Student** |  |  | Cognitive Empathy |  | Emotional Empathy |  | Social Identification |  | Perceived Warmth | |
|  | Emotional Empathy | r | **.632** | *** |  |  |  |  |  |  |
|  |  | p | < .001 |  |  |  |  |  |  |  |
|  | Social Identification | r | .101 |  | **.326** | ** |  |  |  |  |
|  |  | p | .346 |  | .002 |  |  |  |  |  |
|  | Perceived Warmth | r | **.250** | * | **.325** | ** | .168 |  |  |  |
|  |  | p | .018 |  | .002 |  | .115 |  |  |  |
|  | Perceived Competence | r | .165 |  | .138 |  | .051 |  | .178 |  |
|  |  | p | .123 |  | .198 |  | .633 |  | .095 |  |

*Note*. **p* < .05, ***p* < .01, ****p* < .001, *n* = 89 participants.

1. **For positive scenarios**

| **Alcoholic person** |  |  | Cognitive Empathy |  | Emotional Empathy |  | Social Identification |  | Perceived Warmth | |
| --- | --- | --- | --- | --- | --- | --- | --- | --- | --- | --- |
|  | Emotional Empathy | r | **.618** | *** |  |  |  |  |  |  |
|  |  | p | < .001 |  |  |  |  |  |  |  |
|  | Social Identification | r | .056 |  | .026 |  |  |  |  |  |
|  |  | p | .601 |  | .810 |  |  |  |  |  |
|  | Perceived Warmth | r | .178 |  | **.329** | ** | **.258** | * |  |  |
|  |  | p | .096 |  | .002 |  | .015 |  |  |  |
|  | Perceived Competence | r | .024 |  | **.276** | ** | .109 |  | **.606** | *** |
|  |  | p | .826 |  | .009 |  | .310 |  | < .001 |  |
| **Businessperson** |  |  | Cognitive Empathy |  | Emotional Empathy |  | Social Identification |  | Perceived Warmth | |
|  | Emotional Empathy | r | **.601** | *** |  |  |  |  |  |  |
|  |  | p | < .001 |  |  |  |  |  |  |  |
|  | Social Identification | r | .204 |  | .189 |  |  |  |  |  |
|  |  | p | .055 |  | .076 |  |  |  |  |  |
|  | Perceived Warmth | r | .195 |  | **.322** | ** | **.271** | * |  |  |
|  |  | p | .068 |  | .002 |  | .010 |  |  |  |
|  | Perceived Competence | r | .202 |  | .100 |  | .103 |  | -.120 |  |
|  |  | p | .058 |  | .350 |  | .335 |  | .265 |  |
| **Elderly person** |  |  | Cognitive Empathy |  | Emotional Empathy |  | Social Identification |  | Perceived Warmth | |
|  | Emotional Empathy | r | **.646** | *** |  |  |  |  |  |  |
|  |  | p | < .001 |  |  |  |  |  |  |  |
|  | Social Identification | r | .148 |  | .132 |  |  |  |  |  |
|  |  | p | .165 |  | .216 |  |  |  |  |  |
|  | Perceived Warmth | r | **.351** | *** | **.289** | ** | .138 |  |  |  |
|  |  | p | < .001 |  | .006 |  | .196 |  |  |  |
|  | Perceived Competence | r | **.274** | ** | **.326** | ** | .206 |  | **.310** | ** |
|  |  | p | .009 |  | .002 |  | .053 |  | .003 |  |
| **Student** |  |  | Cognitive Empathy |  | Emotional Empathy |  | Social Identification |  | Perceived Warmth | |
|  | Emotional Empathy | r | **.645** | *** |  |  |  |  |  |  |
|  |  | p | < .001 |  |  |  |  |  |  |  |
|  | Social Identification | r | .190 |  | **.332** | ** |  |  |  |  |
|  |  | p | .075 |  | .001 |  |  |  |  |  |
|  | Perceived Warmth | r | **.317** | ** | **.347** | *** | .168 |  |  |  |
|  |  | p | .002 |  | < .001 |  | .115 |  |  |  |
|  | Perceived Competence | r | .179 |  | .142 |  | .051 |  | .178 |  |
|  |  | p | .093 |  | .184 |  | .633 |  | .095 |  |

*Note*. **p* < .05, ***p* < .01, ****p* < .001, *n* = 89 participants.

1. **For negative scenarios**

| **Alcoholic person** |  |  | Cognitive Empathy |  | Emotional Empathy |  | Social Identification |  | Perceived Warmth | |
| --- | --- | --- | --- | --- | --- | --- | --- | --- | --- | --- |
|  | Emotional Empathy | r | **.521** | *** |  |  |  |  |  |  |
|  |  | p | < .001 |  |  |  |  |  |  |  |
|  | Social Identification | r | .021 |  | .024 |  |  |  |  |  |
|  |  | p | .848 |  | .825 |  |  |  |  |  |
|  | Perceived Warmth | r | .050 |  | .179 |  | **.258** | * |  |  |
|  |  | p | .643 |  | .094 |  | .015 |  |  |  |
|  | Perceived Competence | r | -.140 |  | .071 |  | .109 |  | **.606** | *** |
|  |  | p | .190 |  | .507 |  | .310 |  | < .001 |  |
| **Businessperson** |  |  | Cognitive Empathy |  | Emotional Empathy |  | Social Identification |  | Perceived Warmth | |
|  | Emotional Empathy | r | **.447** | *** |  |  |  |  |  |  |
|  |  | p | < .001 |  |  |  |  |  |  |  |
|  | Social Identification | r | .072 |  | .201 |  |  |  |  |  |
|  |  | p | .503 |  | .059 |  |  |  |  |  |
|  | Perceived Warmth | r | -.016 |  | **.327** | ** | **.271** | * |  |  |
|  |  | p | .883 |  | .002 |  | .010 |  |  |  |
|  | Perceived Competence | r | .158 |  | .129 |  | .103 |  | -.120 |  |
|  |  | p | .139 |  | .228 |  | .335 |  | .265 |  |
| **Elderly person** |  |  | Cognitive Empathy |  | Emotional Empathy |  | Social Identification |  | Perceived Warmth | |
|  | Emotional Empathy | r | **.584** | *** |  |  |  |  |  |  |
|  |  | p | < .001 |  |  |  |  |  |  |  |
|  | Social Identification | r | .022 |  | .017 |  |  |  |  |  |
|  |  | p | .837 |  | .878 |  |  |  |  |  |
|  | Perceived Warmth | r | .202 |  | **.209** | * | .138 |  |  |  |
|  |  | p | .058 |  | .049 |  | .196 |  |  |  |
|  | Perceived Competence | r | .206 |  | **.256** | * | .206 |  | **.310** | ** |
|  |  | p | .052 |  | .016 |  | .053 |  | .003 |  |
| **Student** |  |  | Cognitive Empathy |  | Emotional Empathy |  | Social Identification |  | Perceived Warmth | |
|  | Emotional Empathy | r | **.631** | *** |  |  |  |  |  |  |
|  |  | p | < .001 |  |  |  |  |  |  |  |
|  | Social Identification | r | -.024 |  | **.273** | ** |  |  |  |  |
|  |  | p | .824 |  | .010 |  |  |  |  |  |
|  | Perceived Warmth | r | .115 |  | **.252** | * | .168 |  |  |  |
|  |  | p | .282 |  | .017 |  | .115 |  |  |  |
|  | Perceived Competence | r | .110 |  | .113 |  | .051 |  | .178 |  |
|  |  | p | .306 |  | .290 |  | .633 |  | .095 |  |

*Note*. **p* < .05, ***p* < .01, ****p* < .001, *n* = 89 participants.
