# Supplementary material for: Empathic responses to social targets: The influence of warmth and competence perceptions, situational valence, and social identification
Source: PLoS One. 2021 Mar 15;16(3):e0248562. doi: 10.1371/journal.pone.0248562 (PMC7959363; doi:10.1371/journal.pone.0248562)
Supplement: S1 Analysis — (DOCX) [file pone.0248562.s006.docx]

## **S1 Analysis: Ratings of social identification and perceived warmth and competence (manipulation check)**

We calculated three repeated-measures analyses of variance (ANOVAs) with the factor character (student, elderly person, businessperson, alcoholic person), one for each of the following dependent variables: IOS scores and ratings of warmth and competence attributed to the different social targets. All ANOVAs revealed significant effects of character (IOS: (*F*(2.43, 213.85) = 229.86, Greenhouse-Geisser corrected, *p* < .0005; warmth: *F*(3, 264) = 380.35, Greenhouse Geisser corrected, *p* < .0005; competence: (*F*(3, 261) = 389.10, sphericity assumed, *p* < .0001), and subsequent pairwise comparisons were performed. Participants perceived the characters as expected. In line with our expectations, participants identified most strongly with the student character (*M* = 6.0, *SD* = 1.31), followed by the elderly (*M* = 3.5, *SD* = 1.67), the businessperson (*M* = 2.3, *SD* = 1.15), and the alcoholic (*M* = 1.5, *SD* = 0.83) characters. All pairwise comparisons reached significance (*p*s < .0005).

Warm characters were rated as being warmer than cold characters, with the elderly character (*M* = 88.4, *SD* =11.78) receiving higher warmth ratings than the businessperson (*M* = 30.6, *SD* = 17.30, *p* < .0005) and the alcoholic character (*M* = 28.1, *SD* = 17.30, *p* < .0005). The same held for the student (*M* = 75.5, *SD* = 11.60; *p* < .0005, for both the comparison with the businessperson and with the alcoholic person). Although there was no difference in perceived warmth between the two cold characters (*p* = 1.00), there was a difference for the two warm characters. Specifically, the elderly character received greater warmth scores than did the student character (*p* < .0005).

Post hoc tests for competence revealed that, in accordance with our assumptions, low competent characters were rated as less competent than highly competent characters. Yet, the fact that all Bonferroni tests turned out to be significant (*p* < .0005) reflected that there were also differences between the competence ratings attributed to the two cold characters and between those given for the two warm characters. The businessperson (*M* = 87.4, *SD* = 13.41) was perceived as most competent, followed by the student (*M* = 71.8, *SD* = 15.14), the elderly (*M* = 57.1, *SD* = 17.49), and, lastly, the alcoholic (*M* = 13.9, *SD* = 14.40) character.

Because our participants’ warmth and competence ratings revealed significant differences even between those targets that, according to theory, were assumed to be characterized by a similar extent of warmth or competence (i.e., differences in warmth between the elderly and student characters; differences in competence between the businessperson and student character, as well as between the elderly and alcoholic character, a pattern that is not uncommon in research on the SCM; e.g., [1, 2]), we entered those ratings into our primary multilevel models as continuous instead of categorical predictors (see main manuscript). For comparison purposes with earlier investigations based on the SCM model, we additionally conducted multilevel models with warmth and competence as factors (S2 Analysis).

**References**

1. Cikara, M. and S.T. Fiske, *Bounded empathy: Neural responses to outgroup targets' (mis)fortunes.* Journal of cognitive neuroscience, 2011. **23**(12): p. 3791-3803.

2. Cuddy, A.J., M.I. Norton, and S.T. Fiske, *This old stereotype: The pervasiveness and persistence of the elderly stereotype.* Journal of social issues, 2005. **61**(2): p. 267-285.
