# Supplementary material for: Empathic responses to social targets: The influence of warmth and competence perceptions, situational valence, and social identification
Source: PLoS One. 2021 Mar 15;16(3):e0248562. doi: 10.1371/journal.pone.0248562 (PMC7959363; doi:10.1371/journal.pone.0248562)
Supplement: S2 Analysis — (DOCX) [file pone.0248562.s007.docx]

## ***S2 Analysis: Linear mixed models with warmth and competence as categorical predictors.***

## Cognitive empathy (mentalizing, attribution of feelings)

For the model that included all predictors (warmth, competence, valence, IOS), as well as all possible (two-way, three-way, and four-way) interactions as fixed effects, AIC and BIC were 89907.733 and 90137.679, respectively. In this model, the four-way interaction (*p* = .328) and three of the four three-way interactions were nonsignificant (warmth × valence × IOS: *p* = .472; competence × valence × IOS: *p* = .178; warmth × competence × IOS: *p* = .680). After we removed the nonsignificant four-way and three-way interactions, the information criteria (AIC = 89903.325, BIC = 90106.401) of the final linear mixed model (including main effects, all two-way interactions, and the three-way interaction warmth × competence × valence) were both lower than for the full model above. The model also showed significant random slope variances for all three categorical predictors across participants (warmth: LR $\chi^{2}$(4) = 84.911, *p* < .001; competence: LR $\chi^{2}$(4) = 82.202, *p* < .001; valence: LR $\chi^{2}$(4) = 287.484, *p* < .001), as well as for warmth (LR $\chi^{2}$(3) = 144.812, *p* < .001) and competence (LR $\chi^{2}$(3) = 140.473, p < .001) across scenarios.

Concerning the fixed effects, the final model revealed a main effect of warmth (*F*(1, 81.3) = 9.64, *p* = .003) qualified by an interaction of warmth with valence (*F*(1, 44.6) = 8.89, *p* = .005). Similarly, there was a main effect of competence (*F*(1, 52.5) = 7.93, *p* = .007) qualified by an interaction of competence with valence (*F*(1, 34.7) = 5.34, *p* = .027). Both two-way interaction effects were in turn qualified by a significant three-way interaction between warmth, competence, and valence (*F*(1, 11025.2) = 83.59, *p* < .001; Fig S2.1) in predicting cognitive empathy.

For negative situations (Fig S2.1, panel A), similar levels of cognitive empathy resulted for all four characters: warm-incompetent (i.e., elderly person: *M* = 30.7, *SE* = 2.36), warm-competent (i.e., student: *M* = 28.6, *SE* = 2.39), cold-incompetent (i.e., alcoholic person: *M* = 28.6, *SE* = 2.22), cold-competent (i.e., businessperson: *M* = 29.7, *SE* = 2.20). Simple effects analyses showed that none of the conditional effects of warmth (with competence as moderator) or of competence (with warmth as moderator) were significant for negative situations (*p*s 0.077 to 0.407).

**Fig S2.1: Interaction effects between warmth, competence, and valence on ratings of cognitive empathy.** SE = standard error

### Valence = Negative


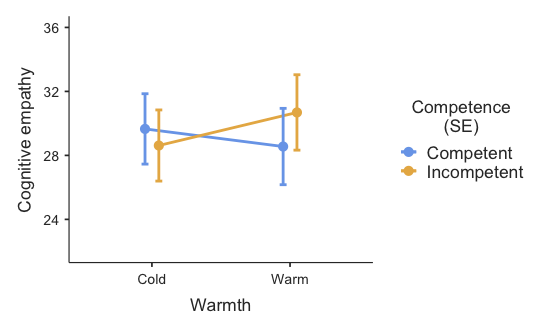


### Valence = Positive


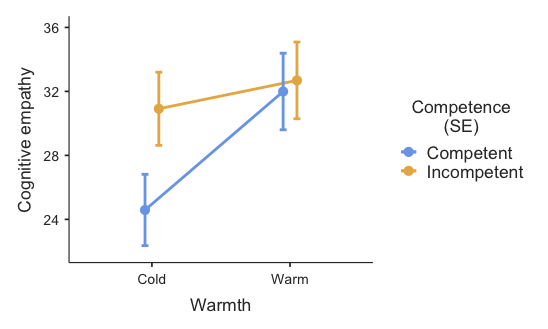


For positive situations (Fig S2.1, panel B), similar levels of cognitive empathy resulted for the warm-incompetent character (i.e., elderly person: *M* = 32.7, *SE* = 2.40), the warm-competent character (i.e., student: *M* = 32.0, *SE* = 2.39), and the cold-incompetent character (i.e., alcoholic person: *M* = 30.9, *SE* = 2.29). By contrast, the cold-competent character was rated lowest on cognitive empathy during positive situations (i.e., businessperson: *M* = 24.6, *SE* = 2.23). Simple effects analyses showed that warmth played a role in cognitive empathy for competent characters (*b* = 7.406, *b_s_* = 0.454, *t*(136.4) = 5.66, *p* < .001), but not for incompetent characters (*b* = 1.772, *b_s_* = 0.108, *t*(87.5) = 1.53, *p* = .131), and competence played a role in cognitive empathy for cold characters (*b* = 6.323, *b_s_* = 0.387, *t*(71.8) = 5.79, *p* < .001), but not for warm characters (*b* = 0.694, *b_s_* = 0.042, *t*(102.3) = 0.58, *p* = .565).

Finally, there was also a main effect of the degree of social identification with the target character (*F*(1, 364.3) = 20.87, *p* < .001). However, this effect was qualified by an interaction of degree of identification with valence (*F*(1, 6933.5) = 6.71, *p* = .010; Fig S2.2, panel A) and with warmth (*F*(1, 942.7) = 14.86, *p* < .001; Fig S2.2, panel B). Cognitive empathy increased more strongly as a function of social identification with the target character during positive situations (*b* = 0.922, *b_s_* = 0.120; *t*(786.1) = 5.22, *p* < .001) than during negative situations (*b* = 0.392, *b_s_* = 0.051; *t*(806.8) = 2.23, *p* = .026). In addition, cognitive empathy increased as a function of social identification for the cold characters (*b* = 1.218, *b_s_* = 0.159; *t*(556.5) = 5.11, *p* < .001), but not for the warm characters (*b* = 0.096, *b_s_* = 0.012; *t*(688.0) = 0.58, *p* = .559).

**Fig S2.2**. **Interaction between social identification and valence (panel A) and between social identification and warmth (panel B) for cognitive empathy.** SE = standard error. IOS = Inclusion of the Other in the Self scale.

1. **Interaction between social identification and valence**


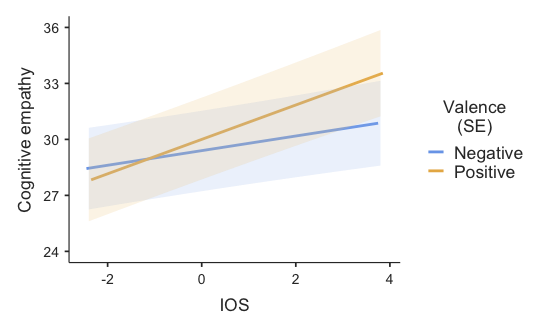


1. **Interaction between social identification and warmth**


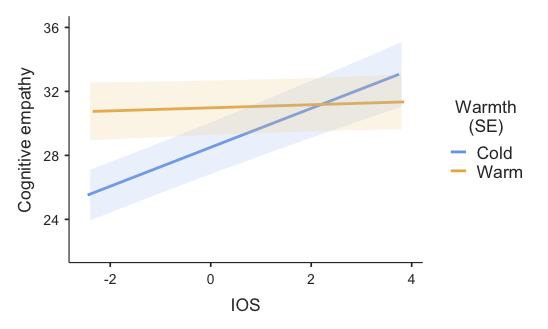


There was no main effect of valence (*F*(1, 31.8) = 0.05, *p* = .821) or statistically significant interaction effects between competence and social identification (F(1, 722.0) = 0.14, *p* = .711) and between warmth and competence (*F*(1, 756.7) = 1.13, *p* = .289).

### Emotional empathy (affective sharing, own feelings)

For the model including all predictors (warmth, competence, valence, IOS), as well as all possible (two-way, three-way, and four-way) interactions as fixed effects, AIC and BIC were 87813.897 and 88047.700, respectively. In this model, the four-way interaction (*p* = .678) and three of the four three-way interactions were nonsignificant (warmth × valence × IOS: *p* = .825; competence × valence × IOS: *p* = .873; warmth × competence × valence: *p* = .524). After we removed the nonsignificant four-way and three-way interactions, the information criteria (AIC =87807.739, BIC = 88014.977) of the final linear mixed model (including main effects, all two-way interactions, and the three-way interaction warmth × competence × IOS) were both lower than for the full model, and the model showed significant random slope variances for all three categorical predictors across participants (warmth: LR $\chi^{2}$(4) = 520.801, *p* < .001; competence: LR $\chi^{2}$(4) = 168.227, *p* < .001; valence: LR $\chi^{2}$(4) = 469.480, *p* < .001), as well as for warmth (LR $\chi^{2}$(3) = 94.974, *p* < .001) and competence (LR $\chi^{2}$(3) = 33.449, *p* < .001) across scenarios.

With regard to fixed effects, the final model revealed a main effect of warmth (*F*(1, 215.6) = 7.44, *p* = .007). Respondents reported higher emotional empathy for warm characters (*M* = 16.6, *SE* = 1.47) than for cold characters (*M* = 14.0, *SE* = 1.47). A main effect of competence (*F*(1, 250.9) = 35.15, *p* < .001) revealed that incompetent characters (*M* = 17.5, *SE* = 1.38) triggered higher levels of emotional empathy than did competent characters (*M* = 13.1, *SE* = 1.27). Descriptively, in terms of the four characters (and thus warmth × competence combinations), respondents displayed the highest levels of empathy for the elderly person (*M* = 18.4, *SE* = 1.50), followed by the alcoholic person (*M* = 16.5, *SE =* 1.48), the student (*M* = 14.8, *SE* = 1.57), and the businessperson (*M* = 11.4, *SE* = 1.20).

There was further a main effect of social identification (*F*(1, 1145.1) = 27.70, *p* < .001) qualified by both an interaction of social identification with warmth (*F*(1, 2041.4) = 22.21, *p* <.001) and a three-way interaction of social identification with warmth and competence (*F*(1, 2404.7) = 5.51, *p* = .019; Fig S2.3).

**Fig S2.3**. **Interaction effects between social identification, warmth, and competence on ratings of emotional empathy.** SE = standard error. IOS = Inclusion of the Other in the Self scale.

### Warmth = Cold


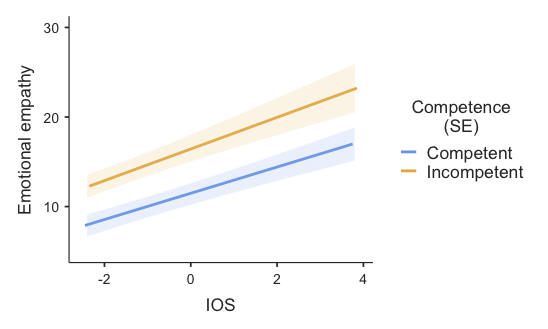


### Warmth = Warm


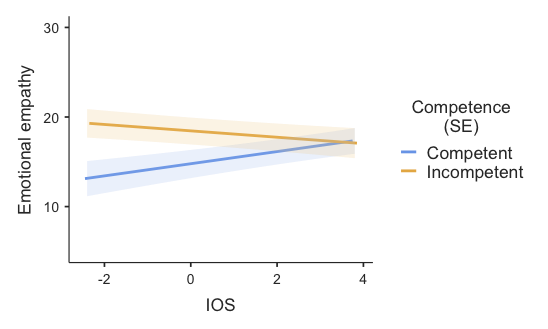


Specifically, respondents reported higher emotional empathy as a function of social identification for cold characters (*b* = 1.616, *b_s_* = 0.228; *t*(1460.1) = 5.96, *p* < .001), but not for warm characters (*b* = 0.161, *b_s_* = 0.023; *t*(1399.8) = 0.91, *p* = .361) when competence was not taken into consideration. Furthermore, simple effects analyses for the three-way interaction showed that emotional empathy for the cold characters increased as a function of social identification, irrespective of the level of competence (incompetent: *b* = 1.766, *b_s_* = 0.249; *t*(1030.9) = 4.17, *p* < .001; competent: *b* = 1.466, *b_s_* = 0.207; *t*(751.5) = 4.86, *p* < .001; Fig S2.3, panel A). For warm characters, by contrast, emotional empathy increased as a function of social identification if the character was competent (*b* = 0.679, *b_s_* = 0.096; *t*(968.3) = 2.51, *p* = .013), but not if the character was incompetent (*b* = -0.356, *b_s_* = -0.050; *t*(958.7) = -1.72, *p* = .087; Fig S2.3, panel B).

There was no main effect of valence (*F*(1, 34.7) = 0.03, *p* = .862) or statistically significant interaction effects between warmth and competence (*F*(1, 1190.7) = 1.40, *p* = .237), between competence and valence (*F*(1, 40.3) = 0.01, *p* = .909), between competence and social identification (*F*(1, 1143.8) = 1.40, *p* = .238), and between valence and social identification (*F*(1, 8995.5) = 2.04, *p* = .153).
